# Physical Activity During Official Match Play in Female Masters Basketball Players: An Accelerometry-Based Study

**DOI:** 10.3390/sports14060230

**Published:** 2026-06-05

**Authors:** Dimitrios Balampanos, Dimitrios Pantazis, Christos Kokkotis, Alexandra Avloniti, Theodoros Stampoulis, Panagiotis Aggelakis, Efstratios Nedeltsos, Georgios Kaltsos, Maria Protopapa, Nikolaos-Orestis Retzepis, Panagiotis Foteinakis, Nikolaos Zaras, Maria Michalopoulou, Athanasios Chatzinikolaou

**Affiliations:** 1 Department of Physical Education and Sport Science, School of Physical Education, Sport Science and Occupational Therapy, Democritus University of Thrace, 69100 Komotini, Greece; dimibala10@phyed.duth.gr (D.B.); dpantazi@phyed.duth.gr (D.P.); pangelak@phyed.duth.gr (P.A.); efstnede@phyed.duth.gr (E.N.); georkalt3@phyed.duth.gr (G.K.); mprotopa@phyed.duth.gr (M.P.); nretzepi@phyed.duth.gr (N.-O.R.); pfotinak@phyed.duth.gr (P.F.); nzaras@phyed.duth.gr (N.Z.); michal@phyed.duth.gr (M.M.); achatzin@phyed.duth.gr (A.C.); 2 Department of Occupational Therapy, School of Physical Education, Sport Science and Occupational Therapy, Democritus University of Thrace, 69100 Komotini, Greece; ckokkoti@ot.duth.gr (C.K.); tstampou@ot.duth.gr (T.S.)

**Keywords:** female masters basketball, physical activity, external load, accelerometry, healthy aging

## Abstract

Background/Objectives: Insufficient physical activity remains a major public health concern among adult women, highlighting the need to identify structured activity contexts that can contribute meaningfully to recommended weekly physical activity levels. Official masters basketball may represent one such context; however, the amount of physical activity accumulated during female masters basketball match play remains insufficiently quantified. This study quantified the physical activity profile of official tournament match play among female masters basketball athletes and described the associated external physical demands. Methods: This observational study included 52 female master basketball athletes aged 37–63 years who competed in a three-day national masters tournament. Match demands were monitored using tri-axial microsensors. Physical activity was classified from processed raw tri-axial acceleration data into intensity zones, and differences in time spent across zones were examined using one-way repeated-measures ANOVA. External load during active play was quantified using total distance, distance across speed zones, accumulated acceleration load (AAL), mechanical load (ML), jump load (JL), and Physio Load. Results: Significant differences were observed across physical-activity intensity zones, with more time accumulated in light physical activity (LPA) and vigorous physical activity (VPA) than in moderate physical activity (MPA), whereas MPA accounted for the least time overall [F (1.98, 101.16) = 47.57, *p* < 0.001, ηp^2^ = 0.48]. Descriptively, moderate-to-vigorous physical activity (MVPA) amounted to 42.78 min, calculated as the sum of MPA (9.41 ± 3.82 min) and VPA (33.37 ± 14.49 min). During active play, athletes covered 59.19 ± 17.26 m·min^−1^, with most distance accumulated in the low- and medium-speed zones and limited very-high-speed running; AAL, ML, and JL averaged 8.32 ± 2.31 AU·min^−1^, 22.35 ± 5.53 AU·min^−1^, and 31.26 ± 28.35 J·min^−1^, respectively. Conclusions: Official female masters basketball appears to provide a meaningful intermittent physical-activity stimulus within a single monitored match exposure and may contribute substantially to weekly aerobic physical-activity accumulation in adult women.

## 1. Introduction

Physical activity is a major determinant of health across adulthood and older age [1,2,3,4,5,6]. In addition to its established role in cardiometabolic health, regular physical activity helps maintain functional capacity, mobility, mental well-being, and independent living [7,8]. Current World Health Organization recommendations advise adults to accumulate 150–300 min of moderate-intensity aerobic physical activity, 75–150 min of vigorous-intensity aerobic physical activity, or an equivalent combination across the week, while older adults are additionally encouraged to engage in varied multicomponent physical activity at moderate or greater intensity on 3 or more days per week to enhance functional capacity and help prevent falls [9,10]. Within this framework, quantifying physical activity, whether derived from organized sport participation or from non-organized forms of daily movement and exercise, is important for understanding how different activity domains may contribute to meeting the World Health Organization’s recommended levels of physical activity.

Organized sport may represent a practical and sustainable means of achieving such exposure. In older adults, participation in sport is commonly motivated by physical and mental health, social connection, and the sense of belonging associated with team-based engagement [11,12,13,14]. Consistent with these motives, participation in sport in later life has also been associated with favorable psychological and social outcomes [15,16,17,18,19]. This is especially relevant in master athletes, who provide a real-world model of continued participation in structured sport across middle and later adulthood [20,21,22]. Compared with sedentary peers, master athletes generally exhibit more favorable physiological function and risk-factor profiles, supporting the view that participation in sport may contribute to the preservation of health and function with advancing age [22,23,24].

Sport participation in later life has received increasing attention because it may offer a socially embedded and sustainable form of physical activity for adults who continue to seek health, competition, and belonging through structured sport [11,16]. However, participation in older adulthood is shaped by health status, previous sport history, social connection, access, and sociodemographic factors, including sex and sport type [16]. Within this context, masters athletes provide a useful real-world model of continued engagement in structured sport across middle and later adulthood [20]. This issue is particularly relevant for women, who remain underrepresented in sport and exercise science research, limiting the extent to which evidence derived mainly from male samples can be generalized to female athletes [25,26]. Female masters athletes therefore represent a relevant but still insufficiently characterized population, especially in intermittent team sports, where physical-activity accumulation and external-load exposure should not be assumed to mirror patterns reported in male cohorts.

Accordingly, the potential health relevance of sport participation should not be inferred from participation alone but should be interpreted in relation to the objectively quantified amount and intensity of physical activity accumulated during play [27,28]. This is particularly important in adult and older populations, in whom review-level evidence indicates that sport participation may improve cardiorespiratory fitness, physical function, mental health, and fat mass, whereas evidence remains more limited or inconclusive for outcomes such as strength, balance, lean mass, and bone mineral density [27,28]. Basketball is a particularly relevant context in this regard, as it is an intermittent, weight-bearing team sport characterized by repeated accelerations, decelerations, changes in direction, and jumping [29,30,31,32]. Beyond its locomotor demands, this activity profile also imposes multidirectional mechanical loading, which may be of musculoskeletal relevance in adult women [33].

Despite the broader relevance of masters sport, the available evidence on official masters basketball remains dominated by male cohorts. In male masters basketball athletes, official match play has been shown to provide a meaningful but age-sensitive amount of physical activity, with Pantazis et al. reporting approximately 27–28 min of moderate-to-vigorous physical activity (MVPA) per game in players aged 35–60 years, compared with 19.6 min in those aged ≥60 years, indicating a shift with advancing age toward less vigorous and more low-intensity activity during play [34]. Earlier work has likewise described the physiological and match-demand profile of official competition primarily in older male players [35,36]. By contrast, substantially fewer studies are available in women, and evidence in female masters basketball athletes remains particularly limited. This is important not only because the literature on female basketball is comparatively sparse and methodologically heterogeneous, but also because women athletes remain underrepresented in sport and exercise research more broadly [25,26,37]. Consequently, the amount of physical activity accumulated during a single official female masters basketball game, together with the accompanying external mechanical demands, remains insufficiently described.

Therefore, the aim of the present study was to quantify the physical activity accumulated during official tournament match play in female masters basketball athletes and to describe the associated external load demands. Specifically, the study examined the distribution of time spent across ENMO-derived physical activity categories throughout the monitored session, as well as selected external load indicators expressed relative to actual active playing time. It was hypothesized that official match play would provide a substantial physical activity stimulus, with meaningful time spent in moderate and vigorous physical activity, alongside external-loading characteristics consistent with a multidirectional, weight-bearing team sport.

## 2. Materials and Methods

### 2.1. Participants

The study used a convenience sample recruited during the 10th National Greek Basketball Maxi Tournament in Komotini, Greece. Data collection was conducted on site, and the sample consisted of 52 women competing in the masters category, aged 37–63 years. Inclusion required at least 10 years of basketball playing experience and regular participation in basketball training during the previous five years, defined as at least two structured sessions per week. Athletes also had to report no musculoskeletal injury, illness, or metabolic disorder that could affect match performance, and no supplement or medication use during the preceding six months.

Because this was a descriptive observational study conducted within a fixed official tournament setting, no formal a priori sample-size calculation was performed. Instead, all eligible athletes who provided consent and met the inclusion criteria were included to maximize sample coverage within the available competitive context. Athletes were excluded if they reported an injury during the preceding six months, a chronic condition with possible effects on physical performance, or medication, alcohol, or tobacco use. Permission for match monitoring was obtained from the participating teams. Before data collection, the athletes were briefed on the study aims, procedures, participation requirements, potential risks, and benefits, and provided written informed consent. The study protocol complied with the Declaration of Helsinki and was approved by the Research Ethics and Deontology Committee of Democritus University of Thrace (Protocol No. DUTH/EHDE/29660/206; approval date: 21 January 2022).

### 2.2. Study Design

Data were collected during four official tournament games. Matches followed International Basketball Federation regulations, with the exception of game duration, as each game consisted of four 8 min periods. Each match was preceded by a 25 min team warm-up that included ball-handling activities, layups, shooting drills, and dynamic stretching. Recording began at the start of the warm-up and continued until the final buzzer. Physical activity and external-load outcomes were calculated over different time windows. Physical activity was analyzed across the full monitored session, from the beginning of the warm-up to the end of the game. In contrast, external-load variables were restricted to actual active playing time, defined as the time each athlete spent on court. Warm-up activity, substitution periods, timeouts, breaks between periods, and halftime were therefore excluded from the external-load calculations. To limit the influence of very short appearances on per-minute external-load estimates, only athletes who accumulated at least 10 min of active playing time were included in the final analysis.

### 2.3. External Load Monitoring

External load was monitored using a Kinexon Perform IMU (KINEXON Sports & Media GmbH, Munich, Germany). Before each match, the sensor was placed in a leather clip case and fixed to the waistband of the athlete’s uniform. Placement was standardized on the right posterior pelvis, level with the iliac crest, so that the unit remained close to the body’s center of mass. Match files were downloaded after play and processed using the KINEXON Sports App/software platform, version 12.0 (KINEXON Sports & Media GmbH, Munich, Germany). The unit contained a tri-axial accelerometer (±16 G; 1 kHz), a tri-axial gyroscope (±4000 deg·s^−1^; 200 Hz), and a tri-axial magnetometer (±16 μT; 100 Hz). The external-load outcomes were total distance per minute, distance per minute within each of the four speed zones, accumulated acceleration load (AAL) per minute, mechanical load (ML) per minute, jump load (JL) per minute, Physio Load per minute, and jumps per minute. Values were normalized to actual active playing time, and arbitrary units were used when required by the device output.

Previous research has examined the validity and reliability of wearable microtechnology and local-positioning systems for external-load monitoring in intermittent team sports. Overall, these systems appear useful for quantifying distance- and speed-related variables, although measurement accuracy may vary according to device model, sampling rate, filtering procedures, movement type, movement intensity, and the specific variable analyzed [38,39]. Kinexon local-positioning technology has also shown acceptable validity against motion-capture reference systems for several indoor team-sport movement tasks, although larger errors may occur during high-acceleration and high-deceleration actions [40]. Therefore, the present external-load outcomes were interpreted as device-derived indicators of active-match demands rather than as interchangeable values across all tracking systems.

AAL was computed from the triaxial acceleration-change signal as √[(Δax)^2^ + (Δay)^2^ + (Δaz)^2^]/100. ML represented the cumulative horizontal acceleration-deceleration output from the x- and *y*-axis signals. JL was computed as body mass × gravitational acceleration × jump height, with gravitational acceleration set at 9.81 m·s^−2^. Physio Load was taken directly from the Kinexon platform and interpreted as a device-derived indicator of physiological intensity during active match participation. The four speed zones were low speed (zone 1, Z1; ≤5.04 km·h^−1^), medium speed (zone 2, Z2; 5.04–10.8 km·h^−1^), high speed (zone 3, Z3; 10.8–18.72 km·h^−1^), and very high speed (zone 4, Z4; >18.72 km·h^−1^). 

### 2.4. Tri-Axial Acceleration Data Processing and Physical Activity Classification

After each match, raw acceleration files were exported from the monitoring system and processed offline using custom Python 3.9 scripts (Python Software Foundation, Wilmington, DE, USA). The three acceleration channels were combined into a single resultant signal by calculating the Euclidean norm across the x, y, and z axes. The gravitational component was then removed using the Euclidean Norm Minus One (ENMO) approach. Values below zero after gravity correction were set to zero, and the retained movement-related acceleration signal was averaged into 1 s epochs before classification.

Physical activity intensity was classified using predefined acceleration cut points linked to metabolic equivalent categories. No physical activity (NPA) was defined as <0.03 g, light physical activity (LPA) as 0.03 to <0.1 g, moderate physical activity (MPA) as 0.1 to <0.4 g, and vigorous physical activity (VPA) as ≥0.4 g. Moderate-to-vigorous physical activity (MVPA) was calculated descriptively as the combined duration of MPA and VPA.

### 2.5. Statistical Analysis

Descriptive statistics were calculated for participant characteristics, physical-activity outcomes, and external-load variables. Continuous data are presented as mean ± standard deviation. Time spent in the four physical-activity intensity zones was analyzed using a one-way repeated-measures analysis of variance, with intensity zone entered as the within-subject factor at four levels: no physical activity, light physical activity, moderate physical activity, and vigorous physical activity.

Moderate-to-vigorous physical activity was analyzed descriptively and was not included as a separate level in the repeated-measures model because it was calculated as the sum of moderate and vigorous physical activity. When a significant main effect was identified, Bonferroni-adjusted pairwise comparisons were performed. Distributional characteristics were inspected using skewness and kurtosis values. Sphericity was evaluated with Mauchly’s test, and Greenhouse–Geisser correction was applied when this assumption was violated. Effect size for the repeated-measures ANOVA was reported as partial eta squared (ηp^2^). Statistical significance was set at *p* < 0.05. All analyses were performed using IBM SPSS Statistics for Windows, version 29.0.2.0 (IBM Corp., Armonk, NY, USA).

## 3. Results

### Participant Characteristics

Participants had a mean age of 47.2 ± 7.0 years, height of 174.0 ± 7.0 cm, and body mass of 70.1 ± 10.0 kg. The descriptive characteristics of the sample are presented in Table 1. The descriptive characteristics of the sample are presented in Table 1.

The active-time external-load profile is presented in Table 2.

**Figure 1:**
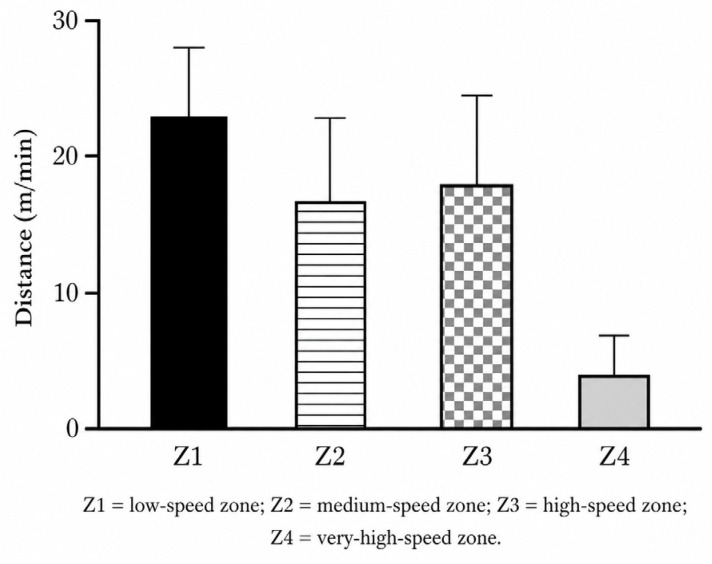
Distance covered across speed zones during active playing time. Z1, low-speed zone (≤5.04 km/h); Z2, medium-speed zone (5.04–10.8 km/h); Z3, high-speed zone (10.8–18.72 km/h); Z4, very-high-speed zone (>18.72 km/h). Data are presented as means ± SD.

Distance covered across speed zones demonstrated that locomotor activity during active play was concentrated predominantly in Z1, Z2, and Z3, whereas Z4 showed the lowest numerical contribution. These findings suggest that the movement demands of official female masters basketball are characterized mainly by low- to high-speed locomotion, with limited contribution from very-high-speed running (Figure 1).

**Figure 2:**
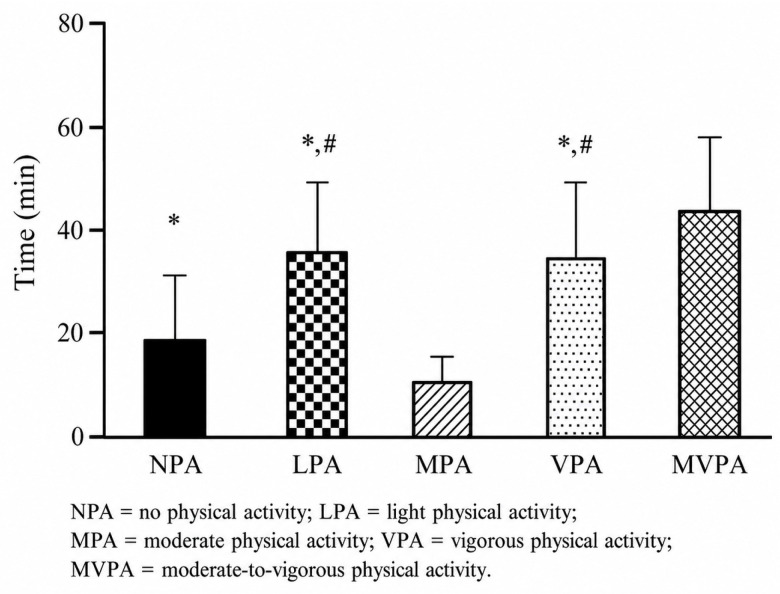
Mean (±SD) time spent across physical-activity intensity categories. The repeated-measures ANOVA included NPA, LPA, MPA, and VPA only. MVPA is displayed descriptively as the sum of MPA and VPA and was not included in the repeated-measures model. * Significantly different from MPA; # significantly different from NPA (Bonferroni-adjusted *p* < 0.05). Error bars represent standard deviations. NPA, no physical activity; LPA, light physical activity; MPA, moderate physical activity; VPA, vigorous physical activity; MVPA, moderate-to-vigorous physical activity.

A one-way repeated-measures ANOVA showed a significant main effect of physical-activity intensity zone on time accumulated across the monitored session [F (1.98, 101.16) = 47.57, *p* < 0.001, ηp^2^ = 0.48]. Bonferroni-adjusted pairwise comparisons showed that NPA differed significantly from LPA and VPA, while MPA was significantly lower than NPA, LPA, and VPA (all *p* < 0.05). LPA and VPA did not differ significantly. MVPA was calculated descriptively as the sum of MPA and VPA and is presented in Figure 2 for interpretive purposes only.

## 4. Discussion

The main finding of the present study was that official female masters basketball provided a meaningful whole-session physical-activity stimulus, characterized by substantial accumulation of moderate-to-vigorous physical activity within an intermittent match-play pattern. Rather than reflecting prolonged time within a stable moderate-intensity range, this profile appears to result from repeated vigorous phases interspersed with lower-intensity movement and recovery periods. This distinction is important because the health relevance of sport participation depends not only on participation itself but also on the amount and intensity of physical activity accumulated within that context [27,28].

A central public health finding of the present study was that a single monitored match exposure accumulated 42.78 min of ENMO-derived moderate-to-vigorous physical activity (MVPA), calculated as the sum of moderate physical activity (MPA; 9.41 min) and vigorous physical activity (VPA; 33.37 min). Relative to current recommendations for adults and older adults, this corresponds to approximately 28.5% of the minimum weekly target of 150 min of moderate-intensity aerobic physical activity [9]. When MPA and VPA were expressed as moderate-equivalent minutes, using the conventional public health equivalence whereby 75–150 min of vigorous-intensity activity is considered equivalent to 150–300 min of moderate-intensity activity, the same monitored exposure corresponded to 76.15 moderate-equivalent minutes, or approximately 50.8% of the lower weekly threshold [9,41]. This does not mean that a single match session fully satisfies the WHO recommendations, because those recommendations also include muscle-strengthening activity and, for older adults with reduced mobility, multicomponent activity emphasizing balance and functional capacity [9]. Nevertheless, the present findings indicate that official female masters basketball can make a substantial contribution to the weekly physical activity targets within a single monitored match session [9,41].

From a broader physical-activity perspective, official female masters basketball may be considered alongside other sport-based exercise options discussed as health-enhancing in adulthood and older age. In recreational futsal, a single 1 h match in sedentary middle-aged men was estimated to expend 634 ± 92 kcal, corresponding to approximately 50% of the weekly physical-activity quantity recommended by the American College of Sports Medicine [42]. Recreational team handball has similarly been characterized as a demanding intermittent exercise mode, with adult untrained men exercising at 82 ± 6% of HRmax on average and spending 47% of match time between 81 and 90% HRmax and 24% above 90% HRmax [43]. Walking football, in contrast, has been profiled as a lower-speed and potentially more accessible team-sport alternative for older adults, with participants aged 66 ± 6 years exercising at 76 ± 6% of HRmax and reporting an RPE of 13 ± 2 [44]. Viewed together, these findings suggest that official female masters basketball may represent a comparatively more demanding sport-based physical activity option than adapted formats such as walking football, while remaining consistent with the broader proposition that intermittent team-sport exposures can meaningfully contribute to health-related activity accumulation [42,43,44]. However, direct comparisons across sports should be approached with caution because differences in game formats, participant characteristics, monitoring tools, and data-reduction procedures substantially affect the resulting estimates of exercise dose.

Specifically in basketball, the present findings are more readily interpretable in light of the limited literature on official masters competition. Pantazis et al. [34] reported age-group MVPA means of 27.46, 27.99, and 19.58 min for male masters basketball athletes, which were numerically lower than the 42.78 min observed in the present female sample. Direct comparison should nevertheless be approached with caution because the male data were age-stratified, whereas the present female data were pooled across match observations [34]. Even so, the current results support the view that official female masters basketball can provide substantial whole-session MVPA exposure while extending the masters-basketball literature to a population that has remained largely unexamined, namely female master athletes during official match play [34]. Beyond the absolute amount of MVPA, the internal structure of the physical-activity profile is also informative. The monitored session was characterized by relatively high LPA and VPA, whereas MPA was markedly lower. This pattern should not be interpreted as anomalous. Basketball is consistently described as an intermittent team sport in which repeated high-intensity actions are embedded within lower-intensity locomotor, positional, and recovery phases rather than accumulated through sustained continuous work [29,30]. Reviews of basketball match play demands likewise emphasize the recurrent accelerations, decelerations, changes in direction, and short high-intensity efforts that shape the sport’s activity profile across competitive settings [29,30]. In the present study, this intermittent structure was likely accentuated by the fact that physical activity was quantified across the entire monitored session, from the start of the warm-up to the end of the match, rather than only during active court time. Accordingly, the relatively large contribution of LPA likely reflects lower-intensity movement and transitional phases across the monitored exposure, whereas the substantial VPA contribution indicates that the session still included repeated vigorous bursts despite the middle-to-later-adulthood profile of the participants [29,30].

That whole-session physical activity pattern becomes clearer when considered alongside the corresponding active-time external load profile. During actual active playing time, athletes covered 59.19 m·min^−1^, with most of the distance accumulated in the low- and medium-speed zones, whereas very-high-speed running remained limited. Accumulated acceleration load averaged 8.32 AU·min^−1^, relative mechanical load 22.35 AU·min^−1^, jump load 31.26 J·min^−1^, and jump frequency 0.21 jumps·min^−1^. Together, these values describe a competition profile characterized primarily by continuous intermittent locomotion, modest exposure to high-speed running, and limited jumping frequency. This interpretation is broadly consistent with official game data in older male basketball players, where Conte et al. [36] described relatively modest external-load intensity compared with younger basketball populations, with lower-intensity classes predominating for accelerations, changes in direction, and jumps [36]. A similar pattern is also evident in the master’s basketball data of Pantazis et al. [34], in which accumulated acceleration load ranged from 6.74 to 11.35 AU·min^−1^, mechanical load from 19.75 to 27.45 AU·min^−1^, total distance from 52.71 to 82.45 m·min^−1^, and total jumps from 0.25 to 0.42 events·min^−1^ across male age groups [34]. The present female values, therefore, lie within the general descriptive range of official masters basketball competition but appear to reflect a comparatively modest vertical-load profile, particularly in relation to jumping frequency [34,36].

Importantly, the present profile should not be interpreted as indicating low overall physical demand. The coexistence of substantial whole-session MVPA with a comparatively modest active-time locomotor and jumping profile suggests that vigorous efforts persist but are expressed intermittently, interspersed with lower-intensity phases. From a practical standpoint, official female masters basketball may therefore provide a meaningful aerobic stimulus without requiring prolonged very-high-speed running or frequent repeated jumping. However, this interpretation should be approached with caution in relation to the broader female basketball literature, which remains methodologically heterogeneous across competitive levels, devices, variables, and reduction procedures [37]. The present findings should therefore be viewed as an age- and competition-specific description of official female masters match play rather than as a direct benchmark against other female basketball populations [37].

From a practical perspective, the present findings suggest that official female masters basketball may provide meaningful moderate-to-vigorous physical-activity exposure despite limited very-high-speed running and relatively low jumping frequency. This may be relevant for adult women seeking a structured, team-based form of sport participation that contributes to weekly physical-activity accumulation. At the same time, coaches and practitioners should recognize the intermittent nature of the activity profile, in which vigorous bursts are interspersed with lower-intensity movement and recovery phases. Individual training history, recovery status, and musculoskeletal readiness should therefore be considered when promoting or programming masters basketball for health- or fitness-oriented participation.

A key methodological consideration is that physical activity was quantified throughout the entire monitored session, whereas external-load variables were normalized only to actual active playing time. These outputs are therefore complementary rather than directly interchangeable. Specifically, the physical-activity findings describe the overall exposure accumulated across the monitored match session, whereas the external-load variables describe the intensity and movement demands of the time actually spent on court. This distinction is important for interpretation because it helps explain why a monitored session may contribute meaningfully to weekly aerobic physical-activity targets while still exhibiting a comparatively modest active-time external-load profile.

Several limitations should be acknowledged. First, the sample was recruited from a single national masters tournament and included four official matches, which may limit the generalizability of the findings to other competitive contexts, tournament formats, or countries. Second, the cross-sectional design describes match-play demands but does not allow conclusions about longitudinal health adaptations. Third, physiological variables such as heart rate, oxygen uptake, or blood lactate were not collected, limiting the interpretation of internal physiological strain. Fourth, the analysis was not stratified by playing position, and position-specific differences in physical activity or external load may therefore have been obscured. Finally, although athletes were screened for recent injury, detailed injury-history stratification was not available.

## 5. Conclusions

Official female masters basketball appears to provide a substantial intermittent physical-activity stimulus during a single monitored match exposure. The large effect observed across physical-activity intensity zones supports the interpretation that match play produces a clearly differentiated intensity profile, with meaningful moderate-to-vigorous physical-activity accumulation. As such, participation may contribute to weekly aerobic physical-activity targets in adulthood and later life. From a practical perspective, these findings support official masters basketball as a viable, organized sport-based physical-activity context for adult women. Future research should examine physiological responses, position-specific demands, and longitudinal health-related adaptations.

## Figures and Tables

**Table 1:** Descriptive characteristics.

Variable	Mean	SD	Min	Max
Age	47.19	6.98	37	63
Height (cm)	174	7.0	160	188
Body Mass (kg)	70.13	10.01	52	95

**Table 2:** Workload profile during active playing time in female masters basketball athletes.

Metric	Mean	SD
Distance (m·min^−1^)	59.19	17.26
AAL (AU·min^−1^)	8.32	2.31
Jump Load (J·min^−1^)	31.26	28.35
Mechanical Load (AU·min^−1^)	22.35	5.53
Physio Load (AU·min^−1^)	9.88	3.31

Data are presented as means ± SD. AAL, accumulated acceleration load; AU, arbitrary units.

## Data Availability

The data used in this study are not publicly available due to privacy and ethical restrictions. Access is restricted to the research team to protect participant confidentiality.
